# Crystal structure of (2*Z*)-2-{(5*Z*)-5-[3-fluoro-2-(4-phenyl­piperidin-1-yl)benzyl­idene]-4-oxo-3-(*p*-tol­yl)-1,3-thia­zolidin-2-yl­idene}-*N*-(*p*-tol­yl)ethane­thio­amide dimethyl sulfoxide monosolvate

**DOI:** 10.1107/S2056989015016850

**Published:** 2015-09-12

**Authors:** Liliya Khamidullina, Konstantin Obydennov, Pavel Slepukhin, Yury Morzherin

**Affiliations:** a Ural Federal University, 19 Mira Str., Ekaterinburg 620002, Russian Federation; b I. Ya. Postovsky Institute of Organic Synthesis, 22 S. Kovalevskoy Str., Ekaterinburg 620990, Russian Federation

**Keywords:** crystal structure, thio­amide, exocyclic double bond, thia­zol­idine, hydrogen bonding

## Abstract

The title compound, C_37_H_34_FN_3_OS_2_·C_2_H_6_OS, was obtained by the Knoevenagel condensation. The thia­zolidine ring is essentially planar (r.m.s. deviation = 0.025 Å) and forms dihedral angles of 4.2 (3), 68.60 (14) and 39.57 (15)° with the attached thio­amide group, *p*-tolyl group benzene ring and fluoro-substituted benzene ring, respectively. The exocyclic double bonds are in a *Z* configuration. In the crystal, the dimethyl sulfoxide solvent mol­ecule is connected to the main mol­ecule *via* an N—H⋯O hydrogen bond. Weak C—H⋯O hydrogen bonds link the components of the structure into a two-dimensional network parallel to (10-1). Weak intra­molecular C—H⋯S hydrogen bonds are also observed. The crystal is an inversion twin with a ratio of twin components 0.78 (2):0.22 (6).

## Related literature   

For non-covalent inter­actions, see: Minkin & Minyaev (2001 ▸); Bjernemose *et al.* (2003 ▸). For the biological activity of thia­zolidines, see: Nazreen *et al.* (2015 ▸); Tripathi *et al.* (2014 ▸). For docking investigations of thia­zolidines, see: Sharma *et al.* (2015 ▸); Miyata *et al.* (2013 ▸). For materials applications of thia­zolidines, see: Matsui *et al.* (2010 ▸). For the synthesis of related compounds, see: Obydennov *et al.* (2014 ▸).
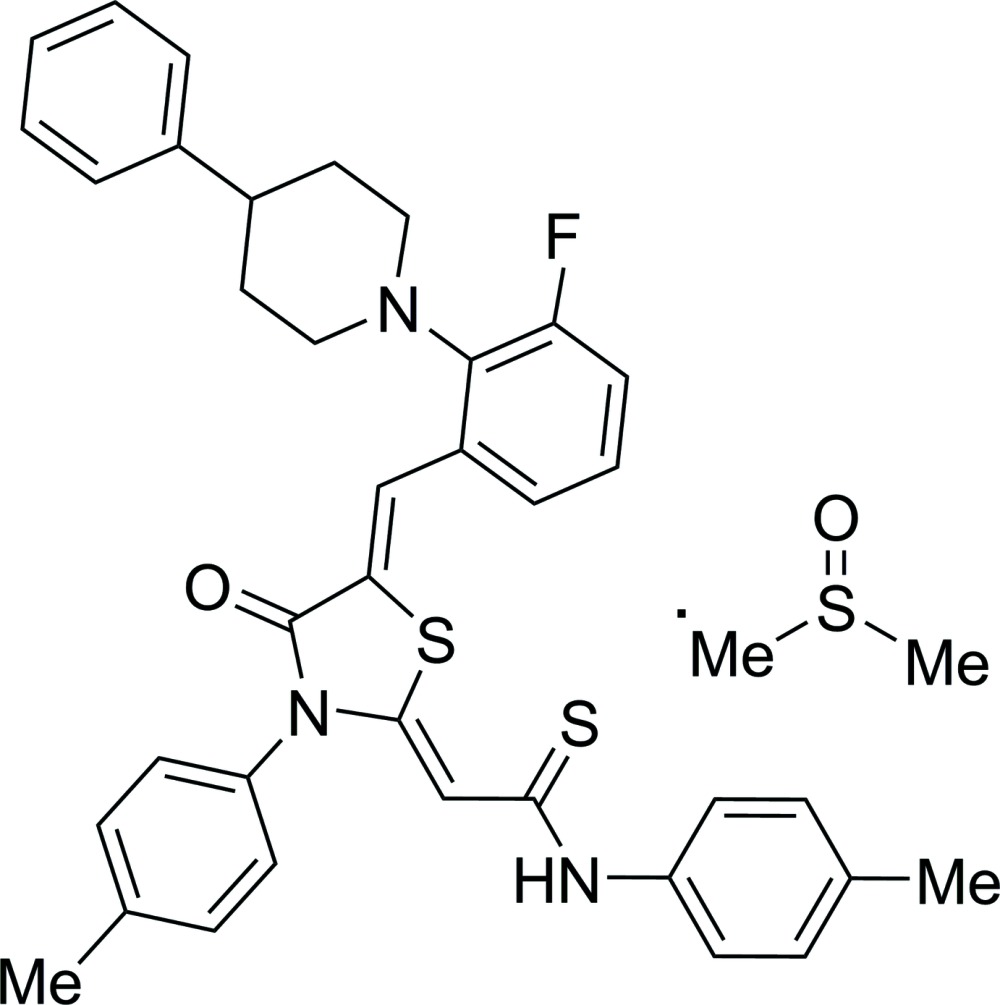


## Experimental   

### Crystal data  

C_37_H_34_FN_3_OS_2_·C_2_H_6_OS
*M* *_r_* = 697.92
Monoclinic, 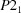

*a* = 9.8539 (5) Å
*b* = 9.8671 (5) Å
*c* = 18.1633 (8) Å
β = 100.578 (5)°
*V* = 1736.01 (14) Å^3^
*Z* = 2
Mo *K*α radiation
μ = 0.26 mm^−1^
*T* = 150 K
0.2 × 0.14 × 0.08 mm

### Data collection  

Agilent Xcalibur Eos diffractometer
Absorption correction: multi-scan (*CrysAlis PRO*; Agilent, 2013 ▸) *T*_min_ = 0.942, *T*_max_ = 1.000
9751 measured reflections
7519 independent reflections
5344 reflections with *I* > 2σ(*I*)
*R*_int_ = 0.029

### Refinement  

*R*[*F* ^2^ > 2σ(*F* ^2^)] = 0.046
*wR*(*F* ^2^) = 0.089
*S* = 1.00
4992 reflections
437 parameters
1 restraint
H-atom parameters constrained
Δρ_max_ = 0.63 e Å^−3^
Δρ_min_ = −0.48 e Å^−3^
Absolute structure: Flack (1983 ▸), 2527 Friedel pairs
Absolute structure parameter: 0.22 (6)

### 

Data collection: *CrysAlis PRO* (Agilent, 2013 ▸); cell refinement: *CrysAlis PRO*; data reduction: *CrysAlis PRO*; program(s) used to solve structure: *SUPERFLIP* (Palatinus & Chapuis, 2007 ▸) and *PLATON* (Spek, 2009 ▸); program(s) used to refine structure: *SHELXS97* (Sheldrick, 2008 ▸); molecular graphics: *OLEX2* (Dolomanov *et al.*, 2009 ▸) and *publCIF* (Westrip, 2010 ▸); software used to prepare material for publication: *OLEX2* and *publCIF*.

## Supplementary Material

Crystal structure: contains datablock(s) I, exp_300. DOI: 10.1107/S2056989015016850/lh5784sup1.cif

Structure factors: contains datablock(s) I. DOI: 10.1107/S2056989015016850/lh5784Isup2.hkl

Supporting information file. DOI: 10.1107/S2056989015016850/lh5784Isup3.cml

**Fig. 1.**
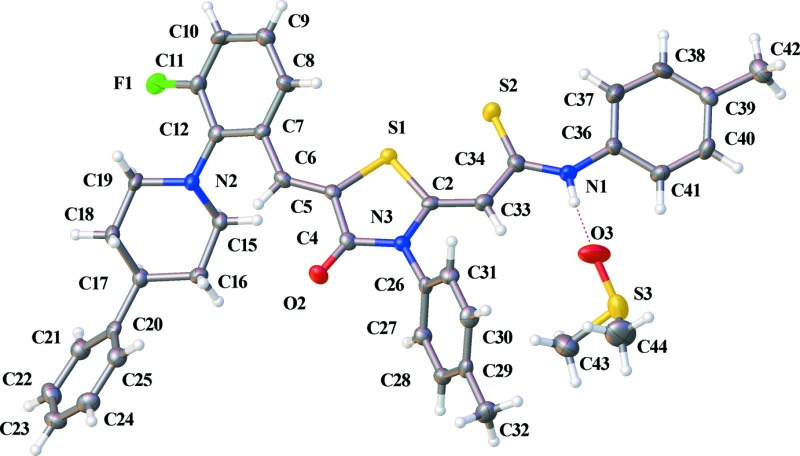
The molecular structure of the title compound showing 50% probability displacement ellipsoids.

. DOI: 10.1107/S2056989015016850/lh5784fig1.tif
The mol­ecular structure of the title compound showing 50% probability displacement ellipsoids.

CCDC reference: 1423184

Additional supporting information:  crystallographic information; 3D view; checkCIF report

## Figures and Tables

**Table 1:** Hydrogen-bond geometry (, )

*D*H*A*	*D*H	H*A*	*D* *A*	*D*H*A*
N1H1O3	0.86	1.97	2.806(4)	164
C8H8S1	0.93	2.68	3.224(3)	118
C19H19*B*O2^i^	0.97	2.53	3.398(4)	149
C32H32*A*O3^ii^	0.96	2.54	3.423(5)	152
C37H37S2	0.93	2.58	3.210(4)	125

Symmetry codes: (i) 
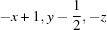
; (ii) 
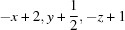
.

## supplementary crystallographic information

### S1. Chemical context

Thia­zolidines are an important class of heteroaromatic compounds and have widespread applications from ranging from pharmaceuticals (Tripathi *et al.*, 2014; Nazreen *et al.*, 2015) to materials (Matsui *et al.*, 2010). The structure determined using X-ray crystallography is useful to perform a docking screen, which is frequently used to predict the binding orientation of potential ligands to their targets in order to in turn predict the affinity and activity of the ligands (Miyata *et al.*, 2013; Sharma *et al.*, 2015). Consequently, we have synthesized the title compound and its crystal structure is presented herein.

### S2. Structural commentary

The molecular structure of the title compound is shown in Fig. 1. The thio­amide group is approximately in the plane of the thia­zolidine ring forming a short S1···S2 contact (Minkin & Minyaev, 2001) with a distance of 2.972 (1) Å. This contact is observed for similar compounds containing a five-membered quasi-ring involving S···S inter­actions (Bjernemose *et al.*, 2003; Obydennov *et al.*, 2014). The *p*-tolyl group benzene ring and thia­zolidine ring form a dihedral angle of 68.60 (14)°. The piperidine ring is in a chair conformation and both the aryl substituents are in equatorial positions. The sum of the bond angles around the thia­zolidine ring N3 atom (360.0°) indicates *sp*^2^ hybridization. The exocyclic double bonds are in a *Z*-configuration. In the crystal, the di­methyl­sulfoxide solvent molecule is connected to the main molecule via an N—H···O hydrogen bond. Weak C—H···O hydrogen bonds link the components of the structure into a two-dimensional network parallel to (101). Weak intra­molecular C—H···S hydrogen bonds are also observed.

### S3. Synthesis and crystallization

\ (2*Z*)-2-[(5*Z*)-5-(3-fluoro-2-(4-phenyl­piperidin-1-yl)benzyl­idene)-\ 4-oxo-3-(*p*-tolyl)-1,3-thia­zolidin-2-yl­idene]-*N*-(*p*-\ tolyl)­ethane­thio­amide was prepared from (2*Z*)-*N*-(4-methyl­phenyl)-2-[3-(4-methyl­phenyl)-4-oxo-1,3-\ thia­zolidin-2-yl­idene]ethane­thio­amide by the Knoevenagel condensation. To a suspension of (2*Z*)-*N*-(4-methyl­phenyl)-2-[3-(4-methyl­phenyl)-4-oxo-1,3-\ thia­zolidin-2-yl­idene]ethane­thio­amide (248 mg, 0.7 mmol) in *n*-butanol (10 ml) were added 3-fluoro-2-(4-phenyl­piperidin-1-yl)benzaldehyde (397 mg, 1.4 mmol) and piperidine (0.06 ml, 0.7 mmol) at room temperature. The mixture was stirred at refluxe for 12 h. After cooling to 255K the crude product was filtered off, recrystallized from ethanol, washed by cooled ethanol and dried *in vacuo*. Yield: 126 mg (29%). ^1^H NMR (400 MHz, DMSO-*d*_6_, *δ*, p.p.m.): 1.68–1.92 (4H, *br.m.*, CH_2_), 2.26 (1H, *s.*, Me), 2.43 (1H, *s.*, Me), 2.70 (1H, *br.m.*, CH), 3.02–3.20 (2H, *br.m.*, CH_2_), 3.20–3.32 (2H, *br.m.*, CH_2_), 6.19 (1H, *s.*, CH=), 7.04–7.66 (15H, *m.*, ArH + CH=), 8.04 (1H, *s.*, ArH), 11.17 (1H, *s.*, NH). Needle-like orange single crystals suitable for X-ray diffraction studies were obtained by slow evaporation of a di­methyl sulfoxide solution of the title compound at room temperature. M.p. 431-433 K.

### S4. Refinement

Hydrogen atoms were placed in calculated positions with C—H = 0.93 - 0.98 Å, N—H = 0.86 Å and included in a riding-model approximation U_iso_(H) = 1.2U_eq_(C,N) or 1.5U_eq_(C) for methyl H atoms.

### Crystal data

**Table:**

C_37_H_34_FN_3_OS_2_·C_2_H_6_OS	*D*_x_ = 1.335 Mg m^−^^3^
*M**_r_* = 697.92	Melting point = 160–158 K
Monoclinic, *P*2_1_	Mo *K*α radiation, λ = 0.71073 Å
*a* = 9.8539 (5) Å	Cell parameters from 2519 reflections
*b* = 9.8671 (5) Å	θ = 2.3–30.2°
*c* = 18.1633 (8) Å	µ = 0.26 mm^−^^1^
β = 100.578 (5)°	*T* = 150 K
*V* = 1736.01 (14) Å^3^	Block, orange
*Z* = 2	0.2 × 0.14 × 0.08 mm
*F*(000) = 736	

### Data collection

**Table:**

Agilent Xcalibur Eos diffractometer	7519 independent reflections
Radiation source: Enhance (Mo) X-ray Source	5344 reflections with *I* > 2σ(*I*)
Graphite monochromator	*R*_int_ = 0.029
Detector resolution: 15.9555 pixels mm^-1^	θ_max_ = 30.8°, θ_min_ = 2.1°
ω scans	*h* = −13→12
Absorption correction: multi-scan (*CrysAlis PRO*; Agilent, 2013)	*k* = −14→14
*T*_min_ = 0.942, *T*_max_ = 1.000	*l* = −25→25
9751 measured reflections	

### Refinement

**Table:**

Refinement on *F*^2^	Secondary atom site location: difference Fourier map
Least-squares matrix: full	Hydrogen site location: inferred from neighbouring sites
*R*[*F*^2^ > 2σ(*F*^2^)] = 0.046	H-atom parameters constrained
*wR*(*F*^2^) = 0.089	*w* = 1/[σ^2^(*F*_o_^2^) + (0.021*P*)^2^] where *P* = (*F*_o_^2^ + 2*F*_c_^2^)/3
*S* = 1.00	(Δ/σ)_max_ < 0.001
4992 reflections	Δρ_max_ = 0.63 e Å^−^^3^
437 parameters	Δρ_min_ = −0.48 e Å^−^^3^
1 restraint	Absolute structure: Flack (1983), 2527 Friedel pairs
Primary atom site location: structure-invariant direct methods	Absolute structure parameter: 0.22 (6)

### Special details

**Table:**

Geometry. All e.s.d.'s (except the e.s.d. in the dihedral angle between two l.s. planes) are estimated using the full covariance matrix. The cell e.s.d.'s are taken into account individually in the estimation of e.s.d.'s in distances, angles and torsion angles; correlations between e.s.d.'s in cell parameters are only used when they are defined by crystal symmetry. An approximate (isotropic) treatment of cell e.s.d.'s is used for estimating e.s.d.'s involving l.s. planes.
Refinement. Refinement of *F*^2^ against ALL reflections. The weighted *R*-factor *wR* and goodness of fit *S* are based on *F*^2^, conventional *R*-factors *R* are based on *F*, with *F* set to zero for negative *F*^2^. The threshold expression of *F*^2^ > σ(*F*^2^) is used only for calculating *R*-factors(gt) *etc*. and is not relevant to the choice of reflections for refinement. *R*-factors based on *F*^2^ are statistically about twice as large as those based on *F*, and *R*-factors based on ALL data will be even larger.

### Fractional atomic coordinates and isotropic or equivalent isotropic displacement parameters (Å^2^)

**Table:**

	*x*	*y*	*z*	*U*_iso_*/*U*_eq_	
S1	0.53305 (8)	0.25119 (8)	0.25856 (4)	0.02326 (18)	
S3	0.80859 (11)	0.72542 (12)	0.55377 (5)	0.0473 (3)	
O2	0.5650 (2)	0.5633 (2)	0.13469 (12)	0.0293 (5)	
F1	0.15403 (19)	−0.03590 (19)	−0.04379 (10)	0.0352 (5)	
C7	0.4153 (3)	0.1509 (3)	0.08448 (17)	0.0211 (7)	
N2	0.2325 (2)	0.2421 (3)	−0.01238 (13)	0.0214 (5)	
N1	0.6889 (3)	0.3429 (3)	0.50937 (15)	0.0294 (7)	
H1	0.7187	0.4250	0.5119	0.035*	
C20	0.0297 (3)	0.5866 (3)	−0.13326 (17)	0.0220 (7)	
C2	0.6082 (3)	0.3936 (3)	0.30804 (18)	0.0201 (7)	
C34	0.6393 (3)	0.3019 (3)	0.43872 (18)	0.0268 (8)	
C39	0.7442 (3)	0.1644 (4)	0.72440 (18)	0.0252 (8)	
C29	0.8222 (3)	0.8738 (3)	0.31806 (17)	0.0245 (8)	
C11	0.2678 (3)	−0.0075 (3)	0.00875 (17)	0.0245 (7)	
C21	−0.1030 (3)	0.6112 (3)	−0.17432 (18)	0.0278 (8)	
H21	−0.1743	0.5523	−0.1692	0.033*	
C12	0.3016 (3)	0.1303 (3)	0.02578 (17)	0.0219 (7)	
C19	0.1557 (3)	0.2225 (3)	−0.08942 (16)	0.0252 (7)	
H19A	0.0671	0.1808	−0.0878	0.030*	
H19B	0.2070	0.1621	−0.1164	0.030*	
C26	0.6885 (3)	0.6261 (3)	0.28185 (16)	0.0192 (7)	
C10	0.3402 (3)	−0.1149 (3)	0.04386 (18)	0.0284 (8)	
H10	0.3145	−0.2032	0.0296	0.034*	
C31	0.8311 (3)	0.6293 (3)	0.30665 (17)	0.0255 (8)	
H31	0.8822	0.5495	0.3109	0.031*	
C33	0.6496 (3)	0.4047 (3)	0.38338 (18)	0.0243 (7)	
H33	0.6886	0.4870	0.4012	0.029*	
C24	0.1034 (4)	0.7911 (3)	−0.1875 (2)	0.0329 (9)	
H24	0.1724	0.8537	−0.1911	0.040*	
O3	0.8366 (3)	0.5875 (3)	0.52409 (16)	0.0588 (8)	
C38	0.7181 (4)	0.0861 (4)	0.6629 (2)	0.0407 (10)	
H38	0.7130	−0.0073	0.6689	0.049*	
C17	0.0588 (3)	0.4582 (3)	−0.08805 (17)	0.0234 (7)	
H17	−0.0305	0.4185	−0.0835	0.028*	
C30	0.8959 (3)	0.7534 (4)	0.32496 (16)	0.0267 (7)	
H30	0.9908	0.7555	0.3422	0.032*	
C28	0.6801 (3)	0.8663 (3)	0.29401 (17)	0.0245 (7)	
H28	0.6283	0.9457	0.2897	0.029*	
C18	0.1332 (3)	0.3556 (3)	−0.12984 (17)	0.0260 (7)	
H18A	0.0791	0.3408	−0.1794	0.031*	
H18B	0.2218	0.3926	−0.1357	0.031*	
C15	0.1606 (3)	0.3354 (3)	0.02973 (17)	0.0237 (7)	
H15A	0.2128	0.3455	0.0802	0.028*	
H15B	0.0706	0.2987	0.0331	0.028*	
C6	0.4630 (3)	0.2886 (3)	0.10419 (17)	0.0209 (7)	
H6	0.4554	0.3498	0.0647	0.025*	
N3	0.6215 (3)	0.5000 (2)	0.25863 (14)	0.0207 (6)	
C36	0.7022 (3)	0.2793 (3)	0.58018 (18)	0.0248 (8)	
C23	−0.0257 (4)	0.8094 (3)	−0.22808 (19)	0.0303 (8)	
H23	−0.0436	0.8830	−0.2604	0.036*	
C22	−0.1303 (3)	0.7198 (4)	−0.22178 (18)	0.0334 (8)	
H22	−0.2185	0.7329	−0.2495	0.040*	
C9	0.4511 (3)	−0.0914 (3)	0.10031 (18)	0.0282 (8)	
H9	0.5014	−0.1635	0.1245	0.034*	
C25	0.1329 (3)	0.6792 (3)	−0.14067 (17)	0.0285 (8)	
H25	0.2220	0.6662	−0.1142	0.034*	
C16	0.1431 (3)	0.4732 (3)	−0.00841 (17)	0.0249 (7)	
H16A	0.2330	0.5112	−0.0107	0.030*	
H16B	0.0961	0.5344	0.0203	0.030*	
C27	0.6140 (3)	0.7448 (4)	0.27641 (16)	0.0236 (7)	
H27	0.5187	0.7427	0.2608	0.028*	
C4	0.5671 (3)	0.4781 (3)	0.18329 (17)	0.0212 (7)	
C41	0.7255 (3)	0.3600 (3)	0.64298 (18)	0.0289 (8)	
H41	0.7270	0.4537	0.6378	0.035*	
C42	0.7674 (3)	0.1067 (4)	0.80330 (18)	0.0376 (9)	
H42A	0.7368	0.0142	0.8016	0.056*	
H42B	0.7162	0.1588	0.8335	0.056*	
H42C	0.8639	0.1106	0.8247	0.056*	
C5	0.5160 (3)	0.3375 (3)	0.17185 (17)	0.0191 (7)	
C37	0.6984 (4)	0.1388 (4)	0.5902 (2)	0.0493 (11)	
H37	0.6829	0.0811	0.5490	0.059*	
C8	0.4869 (3)	0.0401 (3)	0.12070 (18)	0.0273 (8)	
H8	0.5607	0.0556	0.1597	0.033*	
C40	0.7468 (4)	0.3030 (4)	0.71362 (19)	0.0304 (8)	
H40	0.7634	0.3596	0.7552	0.036*	
C43	0.6849 (4)	0.7983 (4)	0.4815 (2)	0.0515 (11)	
H43A	0.7139	0.7859	0.4343	0.077*	
H43B	0.6768	0.8934	0.4910	0.077*	
H43C	0.5972	0.7552	0.4802	0.077*	
C32	0.8930 (4)	1.0061 (3)	0.33599 (19)	0.0376 (9)	
H32A	0.9678	0.9949	0.3776	0.056*	
H32B	0.8285	1.0710	0.3487	0.056*	
H32C	0.9284	1.0377	0.2932	0.056*	
S2	0.56650 (10)	0.15033 (9)	0.41505 (5)	0.0337 (2)	
C44	0.9535 (4)	0.8225 (4)	0.5416 (3)	0.0622 (13)	
H44A	1.0315	0.7976	0.5790	0.093*	
H44B	0.9337	0.9170	0.5464	0.093*	
H44C	0.9738	0.8056	0.4927	0.093*	

### Atomic displacement parameters (Å^2^)

**Table:**

	*U*^11^	*U*^22^	*U*^33^	*U*^12^	*U*^13^	*U*^23^
S1	0.0272 (4)	0.0181 (4)	0.0226 (4)	−0.0025 (4)	−0.0007 (3)	0.0005 (4)
S3	0.0589 (7)	0.0459 (7)	0.0323 (5)	−0.0111 (6)	−0.0045 (4)	0.0061 (5)
O2	0.0355 (14)	0.0227 (13)	0.0269 (13)	−0.0079 (11)	−0.0017 (10)	0.0060 (11)
F1	0.0356 (12)	0.0279 (11)	0.0369 (12)	−0.0098 (9)	−0.0068 (9)	−0.0063 (10)
C7	0.0237 (16)	0.0191 (16)	0.0204 (16)	0.0005 (15)	0.0037 (13)	−0.0021 (15)
N2	0.0234 (13)	0.0218 (14)	0.0178 (13)	0.0016 (13)	0.0006 (10)	−0.0025 (13)
N1	0.0438 (18)	0.0172 (15)	0.0239 (15)	−0.0033 (13)	−0.0025 (13)	−0.0005 (13)
C20	0.0260 (17)	0.0236 (18)	0.0174 (16)	0.0007 (15)	0.0063 (13)	−0.0060 (15)
C2	0.0161 (15)	0.0152 (16)	0.0278 (18)	0.0022 (13)	0.0010 (13)	−0.0025 (15)
C34	0.0283 (18)	0.0236 (17)	0.0261 (19)	0.0015 (15)	−0.0019 (14)	0.0015 (16)
C39	0.0192 (16)	0.030 (2)	0.0256 (18)	0.0035 (16)	0.0018 (13)	0.0053 (17)
C29	0.0332 (19)	0.0246 (19)	0.0156 (16)	−0.0063 (15)	0.0038 (14)	−0.0011 (16)
C11	0.0261 (18)	0.0248 (19)	0.0214 (17)	−0.0067 (15)	0.0009 (14)	−0.0031 (16)
C21	0.0260 (18)	0.028 (2)	0.0291 (19)	−0.0013 (15)	0.0045 (14)	−0.0018 (16)
C12	0.0230 (16)	0.0230 (18)	0.0202 (16)	0.0000 (14)	0.0056 (13)	−0.0017 (15)
C19	0.0303 (17)	0.0243 (19)	0.0187 (16)	0.0038 (15)	−0.0013 (13)	−0.0080 (16)
C26	0.0219 (16)	0.0203 (18)	0.0142 (15)	−0.0052 (14)	0.0000 (12)	0.0002 (14)
C10	0.039 (2)	0.0132 (16)	0.033 (2)	−0.0067 (15)	0.0070 (16)	−0.0013 (16)
C31	0.0233 (17)	0.0248 (19)	0.0282 (18)	0.0055 (15)	0.0043 (14)	0.0000 (16)
C33	0.0238 (18)	0.0196 (17)	0.0277 (18)	−0.0025 (14)	0.0001 (14)	−0.0026 (15)
C24	0.034 (2)	0.027 (2)	0.039 (2)	−0.0075 (16)	0.0115 (17)	0.0002 (18)
O3	0.0551 (18)	0.0225 (15)	0.086 (2)	−0.0061 (13)	−0.0216 (15)	−0.0003 (15)
C38	0.058 (3)	0.0203 (19)	0.036 (2)	0.0052 (18)	−0.0114 (18)	0.0069 (18)
C17	0.0219 (17)	0.0264 (18)	0.0206 (17)	0.0001 (15)	0.0007 (13)	−0.0021 (16)
C30	0.0173 (15)	0.033 (2)	0.0284 (17)	−0.0060 (16)	0.0008 (12)	−0.0008 (18)
C28	0.0293 (19)	0.0207 (18)	0.0222 (17)	−0.0009 (15)	0.0011 (14)	0.0017 (15)
C18	0.0311 (18)	0.0282 (19)	0.0170 (16)	0.0015 (15)	−0.0004 (14)	−0.0032 (15)
C15	0.0258 (18)	0.0267 (19)	0.0183 (16)	0.0031 (15)	0.0035 (13)	−0.0015 (15)
C6	0.0193 (16)	0.0212 (18)	0.0224 (17)	0.0007 (13)	0.0038 (13)	0.0057 (15)
N3	0.0210 (13)	0.0174 (14)	0.0221 (14)	−0.0006 (11)	−0.0004 (11)	0.0001 (12)
C36	0.0294 (18)	0.0213 (19)	0.0208 (17)	−0.0012 (14)	−0.0025 (13)	0.0010 (15)
C23	0.033 (2)	0.0235 (18)	0.035 (2)	0.0049 (16)	0.0070 (16)	0.0063 (16)
C22	0.0300 (18)	0.039 (2)	0.0292 (18)	0.0072 (17)	0.0002 (14)	0.0059 (19)
C9	0.033 (2)	0.0239 (19)	0.0266 (19)	−0.0006 (16)	0.0029 (15)	0.0031 (16)
C25	0.0259 (18)	0.030 (2)	0.0282 (18)	0.0019 (15)	0.0018 (14)	−0.0015 (17)
C16	0.0289 (18)	0.0191 (17)	0.0261 (18)	0.0022 (15)	0.0034 (14)	−0.0020 (15)
C27	0.0194 (15)	0.0254 (17)	0.0245 (16)	−0.0014 (16)	0.0002 (12)	0.0009 (17)
C4	0.0154 (15)	0.0242 (18)	0.0226 (17)	−0.0004 (14)	0.0000 (13)	0.0011 (16)
C41	0.039 (2)	0.0189 (18)	0.0287 (19)	−0.0034 (16)	0.0064 (16)	−0.0031 (16)
C42	0.035 (2)	0.042 (2)	0.036 (2)	0.0032 (17)	0.0043 (17)	0.009 (2)
C5	0.0170 (15)	0.0185 (17)	0.0212 (17)	0.0024 (13)	0.0022 (12)	0.0019 (14)
C37	0.082 (3)	0.030 (2)	0.029 (2)	0.011 (2)	−0.009 (2)	−0.008 (2)
C8	0.0299 (19)	0.0206 (18)	0.0283 (19)	−0.0017 (15)	−0.0030 (15)	0.0034 (16)
C40	0.040 (2)	0.0290 (19)	0.0216 (18)	−0.0070 (17)	0.0050 (15)	−0.0071 (17)
C43	0.064 (3)	0.041 (3)	0.047 (3)	0.011 (2)	0.001 (2)	0.008 (2)
C32	0.048 (2)	0.029 (2)	0.035 (2)	−0.0099 (17)	0.0035 (18)	−0.0025 (18)
S2	0.0512 (6)	0.0236 (5)	0.0245 (5)	−0.0075 (5)	0.0020 (4)	−0.0012 (4)
C44	0.060 (3)	0.044 (3)	0.075 (3)	−0.020 (2)	−0.006 (2)	0.002 (3)

### Geometric parameters (Å, º)

**Table:**

S1—C2	1.757 (3)	C24—H24	0.9300
S1—C5	1.771 (3)	C38—C37	1.399 (5)
S3—O3	1.508 (3)	C38—H38	0.9300
S3—C44	1.766 (4)	C17—C18	1.531 (4)
S3—C43	1.771 (4)	C17—C16	1.537 (4)
O2—C4	1.217 (3)	C17—H17	0.9800
F1—C11	1.361 (3)	C30—H30	0.9300
C7—C8	1.398 (4)	C28—C27	1.374 (4)
C7—C12	1.413 (4)	C28—H28	0.9300
C7—C6	1.460 (4)	C18—H18A	0.9700
N2—C12	1.410 (4)	C18—H18B	0.9700
N2—C15	1.461 (4)	C15—C16	1.521 (4)
N2—C19	1.476 (3)	C15—H15A	0.9700
N1—C34	1.349 (4)	C15—H15B	0.9700
N1—C36	1.416 (4)	C6—C5	1.334 (4)
N1—H1	0.8600	C6—H6	0.9300
C20—C25	1.392 (4)	N3—C4	1.392 (4)
C20—C21	1.402 (4)	C36—C41	1.376 (4)
C20—C17	1.509 (4)	C36—C37	1.400 (5)
C2—C33	1.358 (4)	C23—C22	1.378 (4)
C2—N3	1.402 (4)	C23—H23	0.9300
C34—C33	1.444 (4)	C22—H22	0.9300
C34—S2	1.680 (3)	C9—C8	1.377 (4)
C39—C38	1.344 (5)	C9—H9	0.9300
C39—C40	1.382 (4)	C25—H25	0.9300
C39—C42	1.520 (4)	C16—H16A	0.9700
C29—C30	1.387 (4)	C16—H16B	0.9700
C29—C28	1.390 (4)	C27—H27	0.9300
C29—C32	1.487 (4)	C4—C5	1.477 (4)
C11—C10	1.368 (4)	C41—C40	1.381 (4)
C11—C12	1.420 (4)	C41—H41	0.9300
C21—C22	1.370 (4)	C42—H42A	0.9600
C21—H21	0.9300	C42—H42B	0.9600
C19—C18	1.501 (4)	C42—H42C	0.9600
C19—H19A	0.9700	C37—H37	0.9300
C19—H19B	0.9700	C8—H8	0.9300
C26—C27	1.376 (4)	C40—H40	0.9300
C26—C31	1.395 (4)	C43—H43A	0.9600
C26—N3	1.436 (4)	C43—H43B	0.9600
C10—C9	1.374 (4)	C43—H43C	0.9600
C10—H10	0.9300	C32—H32A	0.9600
C31—C30	1.393 (5)	C32—H32B	0.9600
C31—H31	0.9300	C32—H32C	0.9600
C33—H33	0.9300	C44—H44A	0.9600
C24—C23	1.361 (5)	C44—H44B	0.9600
C24—C25	1.391 (4)	C44—H44C	0.9600
			
C2—S1—C5	91.86 (15)	C19—C18—C17	112.1 (2)
O3—S3—C44	103.86 (19)	C19—C18—H18A	109.2
O3—S3—C43	104.35 (17)	C17—C18—H18A	109.2
C44—S3—C43	98.6 (2)	C19—C18—H18B	109.2
C8—C7—C12	120.3 (3)	C17—C18—H18B	109.2
C8—C7—C6	120.0 (3)	H18A—C18—H18B	107.9
C12—C7—C6	119.6 (3)	N2—C15—C16	110.5 (2)
C12—N2—C15	118.2 (2)	N2—C15—H15A	109.6
C12—N2—C19	118.7 (3)	C16—C15—H15A	109.6
C15—N2—C19	111.4 (2)	N2—C15—H15B	109.6
C34—N1—C36	133.5 (3)	C16—C15—H15B	109.6
C34—N1—H1	113.3	H15A—C15—H15B	108.1
C36—N1—H1	113.3	C5—C6—C7	128.0 (3)
C25—C20—C21	117.5 (3)	C5—C6—H6	116.0
C25—C20—C17	122.3 (3)	C7—C6—H6	116.0
C21—C20—C17	120.0 (3)	C4—N3—C2	116.7 (3)
C33—C2—N3	122.6 (3)	C4—N3—C26	119.8 (3)
C33—C2—S1	126.9 (2)	C2—N3—C26	123.5 (3)
N3—C2—S1	110.6 (2)	C41—C36—C37	118.0 (3)
N1—C34—C33	113.1 (3)	C41—C36—N1	118.0 (3)
N1—C34—S2	125.0 (2)	C37—C36—N1	123.9 (3)
C33—C34—S2	121.9 (3)	C24—C23—C22	120.6 (3)
C38—C39—C40	117.2 (3)	C24—C23—H23	119.7
C38—C39—C42	122.7 (3)	C22—C23—H23	119.7
C40—C39—C42	120.0 (3)	C21—C22—C23	119.3 (3)
C30—C29—C28	117.6 (3)	C21—C22—H22	120.3
C30—C29—C32	121.0 (3)	C23—C22—H22	120.3
C28—C29—C32	121.4 (3)	C10—C9—C8	119.3 (3)
F1—C11—C10	117.3 (3)	C10—C9—H9	120.4
F1—C11—C12	118.7 (3)	C8—C9—H9	120.4
C10—C11—C12	124.0 (3)	C24—C25—C20	120.4 (3)
C22—C21—C20	121.8 (3)	C24—C25—H25	119.8
C22—C21—H21	119.1	C20—C25—H25	119.8
C20—C21—H21	119.1	C15—C16—C17	109.8 (3)
N2—C12—C7	120.2 (3)	C15—C16—H16A	109.7
N2—C12—C11	124.7 (3)	C17—C16—H16A	109.7
C7—C12—C11	115.1 (3)	C15—C16—H16B	109.7
N2—C19—C18	110.7 (3)	C17—C16—H16B	109.7
N2—C19—H19A	109.5	H16A—C16—H16B	108.2
C18—C19—H19A	109.5	C28—C27—C26	120.1 (3)
N2—C19—H19B	109.5	C28—C27—H27	119.9
C18—C19—H19B	109.5	C26—C27—H27	119.9
H19A—C19—H19B	108.1	O2—C4—N3	123.9 (3)
C27—C26—C31	119.8 (3)	O2—C4—C5	125.9 (3)
C27—C26—N3	120.4 (3)	N3—C4—C5	110.1 (3)
C31—C26—N3	119.7 (3)	C36—C41—C40	120.5 (3)
C11—C10—C9	119.5 (3)	C36—C41—H41	119.7
C11—C10—H10	120.3	C40—C41—H41	119.7
C9—C10—H10	120.3	C39—C42—H42A	109.5
C30—C31—C26	119.2 (3)	C39—C42—H42B	109.5
C30—C31—H31	120.4	H42A—C42—H42B	109.5
C26—C31—H31	120.4	C39—C42—H42C	109.5
C2—C33—C34	126.7 (3)	H42A—C42—H42C	109.5
C2—C33—H33	116.6	H42B—C42—H42C	109.5
C34—C33—H33	116.6	C6—C5—C4	122.3 (3)
C23—C24—C25	120.4 (3)	C6—C5—S1	127.0 (2)
C23—C24—H24	119.8	C4—C5—S1	110.6 (2)
C25—C24—H24	119.8	C38—C37—C36	119.3 (3)
C39—C38—C37	122.9 (3)	C38—C37—H37	120.4
C39—C38—H38	118.6	C36—C37—H37	120.4
C37—C38—H38	118.6	C9—C8—C7	121.9 (3)
C20—C17—C18	110.3 (2)	C9—C8—H8	119.1
C20—C17—C16	116.6 (3)	C7—C8—H8	119.1
C18—C17—C16	107.7 (2)	C41—C40—C39	122.1 (3)
C20—C17—H17	107.3	C41—C40—H40	119.0
C18—C17—H17	107.3	C39—C40—H40	119.0
C16—C17—H17	107.3	C29—C32—H32A	109.5
C29—C30—C31	121.5 (3)	C29—C32—H32B	109.5
C29—C30—H30	119.3	H32A—C32—H32B	109.5
C31—C30—H30	119.3	C29—C32—H32C	109.5
C27—C28—C29	121.8 (3)	H32A—C32—H32C	109.5
C27—C28—H28	119.1	H32B—C32—H32C	109.5
C29—C28—H28	119.1		
			
C5—S1—C2—C33	178.6 (3)	C33—C2—N3—C4	−176.1 (3)
C5—S1—C2—N3	−1.4 (2)	S1—C2—N3—C4	3.9 (3)
C36—N1—C34—C33	−179.1 (3)	C33—C2—N3—C26	4.7 (4)
C36—N1—C34—S2	3.2 (5)	S1—C2—N3—C26	−175.3 (2)
C25—C20—C21—C22	1.8 (4)	C27—C26—N3—C4	66.9 (4)
C17—C20—C21—C22	−173.5 (3)	C31—C26—N3—C4	−110.0 (3)
C15—N2—C12—C7	−64.4 (4)	C27—C26—N3—C2	−113.9 (3)
C19—N2—C12—C7	155.6 (3)	C31—C26—N3—C2	69.2 (4)
C15—N2—C12—C11	117.2 (3)	C34—N1—C36—C41	−164.0 (3)
C19—N2—C12—C11	−22.8 (4)	C34—N1—C36—C37	18.6 (6)
C8—C7—C12—N2	−177.8 (3)	C25—C24—C23—C22	2.0 (5)
C6—C7—C12—N2	−1.4 (4)	C20—C21—C22—C23	−1.6 (5)
C8—C7—C12—C11	0.7 (4)	C24—C23—C22—C21	−0.3 (5)
C6—C7—C12—C11	177.1 (3)	C11—C10—C9—C8	0.3 (5)
F1—C11—C12—N2	−5.7 (4)	C23—C24—C25—C20	−1.8 (5)
C10—C11—C12—N2	176.6 (3)	C21—C20—C25—C24	−0.1 (4)
F1—C11—C12—C7	175.8 (2)	C17—C20—C25—C24	175.1 (3)
C10—C11—C12—C7	−1.9 (5)	N2—C15—C16—C17	59.9 (3)
C12—N2—C19—C18	−159.8 (2)	C20—C17—C16—C15	178.9 (2)
C15—N2—C19—C18	57.7 (3)	C18—C17—C16—C15	−56.6 (3)
F1—C11—C10—C9	−176.3 (3)	C29—C28—C27—C26	−0.5 (4)
C12—C11—C10—C9	1.4 (5)	C31—C26—C27—C28	1.2 (4)
C27—C26—C31—C30	−0.5 (4)	N3—C26—C27—C28	−175.7 (3)
N3—C26—C31—C30	176.4 (3)	C2—N3—C4—O2	176.2 (3)
N3—C2—C33—C34	179.5 (3)	C26—N3—C4—O2	−4.6 (4)
S1—C2—C33—C34	−0.6 (5)	C2—N3—C4—C5	−4.8 (3)
N1—C34—C33—C2	−179.8 (3)	C26—N3—C4—C5	174.5 (2)
S2—C34—C33—C2	−2.0 (4)	C37—C36—C41—C40	1.1 (5)
C40—C39—C38—C37	2.0 (5)	N1—C36—C41—C40	−176.5 (3)
C42—C39—C38—C37	−179.2 (3)	C7—C6—C5—C4	176.7 (3)
C25—C20—C17—C18	−73.6 (4)	C7—C6—C5—S1	−4.7 (5)
C21—C20—C17—C18	101.5 (3)	O2—C4—C5—C6	1.3 (5)
C25—C20—C17—C16	49.6 (4)	N3—C4—C5—C6	−177.7 (3)
C21—C20—C17—C16	−135.3 (3)	O2—C4—C5—S1	−177.6 (2)
C28—C29—C30—C31	1.7 (4)	N3—C4—C5—S1	3.5 (3)
C32—C29—C30—C31	−178.5 (3)	C2—S1—C5—C6	−179.9 (3)
C26—C31—C30—C29	−0.9 (4)	C2—S1—C5—C4	−1.2 (2)
C30—C29—C28—C27	−1.0 (4)	C39—C38—C37—C36	−1.7 (6)
C32—C29—C28—C27	179.2 (3)	C41—C36—C37—C38	0.0 (6)
N2—C19—C18—C17	−56.4 (3)	N1—C36—C37—C38	177.4 (3)
C20—C17—C18—C19	−176.0 (3)	C10—C9—C8—C7	−1.4 (5)
C16—C17—C18—C19	55.8 (3)	C12—C7—C8—C9	0.9 (5)
C12—N2—C15—C16	157.4 (3)	C6—C7—C8—C9	−175.5 (3)
C19—N2—C15—C16	−59.9 (3)	C36—C41—C40—C39	−0.7 (6)
C8—C7—C6—C5	−38.0 (5)	C38—C39—C40—C41	−0.8 (5)
C12—C7—C6—C5	145.6 (3)	C42—C39—C40—C41	−179.7 (3)

### Hydrogen-bond geometry (Å, º)

**Table:**

*D*—H···*A*	*D*—H	H···*A*	*D*···*A*	*D*—H···*A*
N1—H1···O3	0.86	1.97	2.806 (4)	164
C8—H8···S1	0.93	2.68	3.224 (3)	118
C19—H19*B*···O2^i^	0.97	2.53	3.398 (4)	149
C32—H32*A*···O3^ii^	0.96	2.54	3.423 (5)	152
C37—H37···S2	0.93	2.58	3.210 (4)	125

Symmetry codes: (i) −*x*+1, *y*−1/2, −*z*; (ii) −*x*+2, *y*+1/2, −*z*+1.
