# Supplementary figures and images for: Crystal structure of (2Z)-2-{(5Z)-5-[3-fluoro-2-(4-phenyl­piperidin-1-yl)benzyl­idene]-4-oxo-3-(p-tol­yl)-1,3-thia­zolidin-2-yl­idene}-N-(p-tol­yl)ethane­thio­amide dimethyl sulfoxide monosolvate
Source: Acta Crystallogr E Crystallogr Commun. 2015 Sep 12;71(Pt 10):o745–6. doi: 10.1107/S2056989015016850 (PMC4647368; doi:10.1107/S2056989015016850)

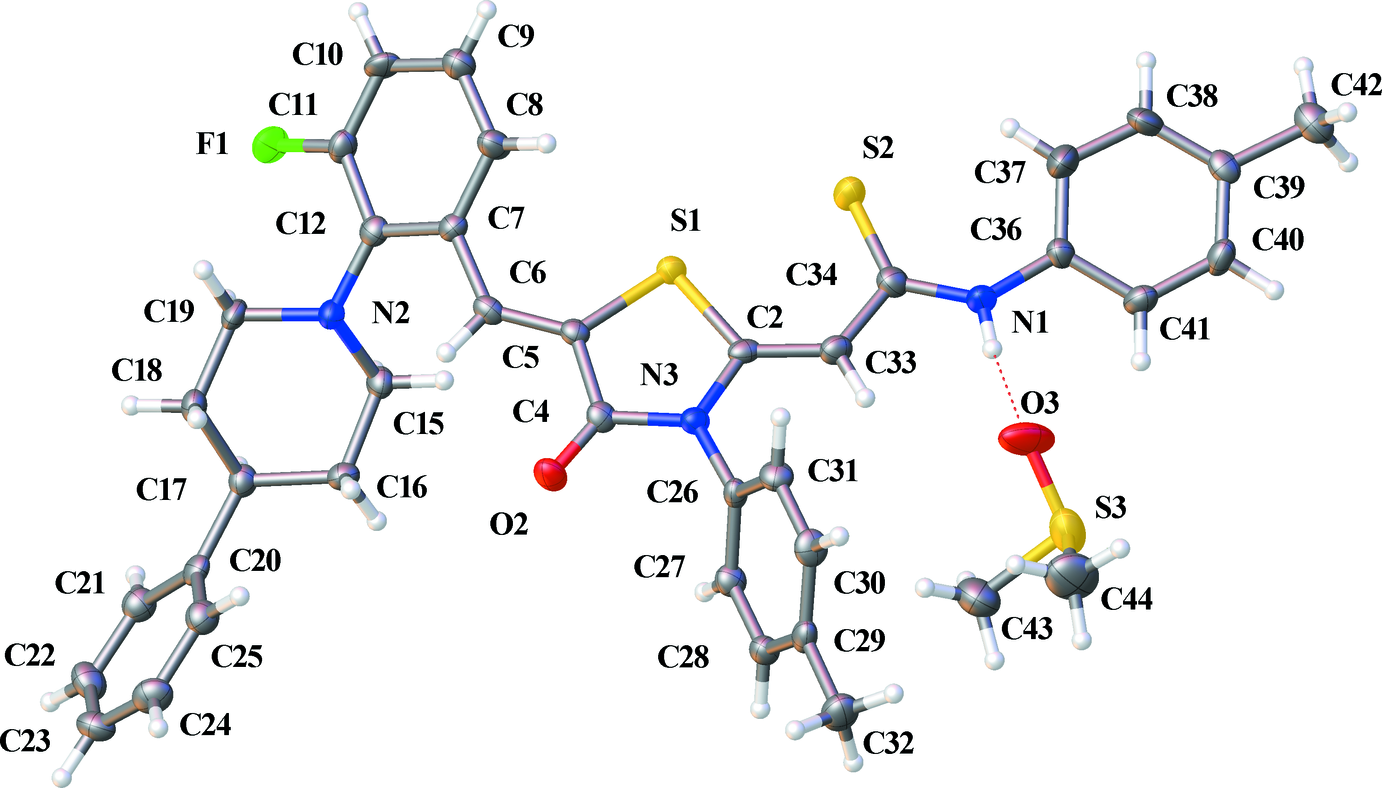

Supplement: Supplementary file 4 [file e-71-0o745-fig1.tif]
